# Prediction of response to anti-TNFα using integrative computational approaches in Crohn’s disease—Needle in a haystack or a promising biomarker?

**DOI:** 10.1016/j.xcrm.2024.101424

**Published:** 2024-02-20

**Authors:** Fatima Zulqarnain, Sana Syed

**Affiliations:** 1 Division of Pediatric Gastroenterology, Hepatology, and Nutrition, Department of Pediatrics, School of Medicine, University of Virginia, Charlottesville, VA, USA

## Abstract

In the January issue of *Cell Reports Medicine*, Gerassy-Vainberg et al.^1^ demonstrate the utility of integrative methods to reveal molecular mechanisms associated with anti-tumor necrosis factor-alpha therapy response in patients with inflammatory conditions.

## Teaser

In the January issue of *Cell Reports Medicine*, Gerassy-Vainberg et al. demonstrate the utility of integrative methods to reveal molecular mechanisms associated with anti-tumor necrosis factor-alpha therapy response in patients with inflammatory conditions.

## Main text

Tumor necrosis factor-alpha (TNF-α) is a proinflammatory cytokine involved in the induction and maintenance of inflammation. While biologic therapies targeting tumor necrosis factor-alpha (anti-TNFα) are the current mainstay in both inflammatory bowel disease (IBD) and various autoimmune conditions, there is no “gold standard” for the assessment of anti-TNFα therapeutic efficacy. Therapeutic decision-making is based on disease symptoms and endoscopic activity, histopathology, and routine laboratory measurements in patients with IBD. Regular anti-TNFα therapy has been shown to increase the likelihood of clinical remission and mucosal healing; however, 20%–40% of patients fail to respond to anti-TNFα therapy either initially or within 12 months of beginning treatment, with non-response being associated with more severe disease progression.^2^^,^^3^^,^^4^ With anti-TNFα therapy rapidly becoming the standard first-line treatment, particularly in severe Crohn’s disease (CD), developing reliable biomarkers of treatment response would augment personalized clinical decision-making by allowing for the early prediction of patient response to anti-TNFα therapy.^5^^,^^6^

High-throughput biomedical data generation has risen meteorically in the last decade. In their recent paper published at *Cell Reports Medicine*, Gerassy-Vainberg et al.^1^ highlight monocytic expression of the RAC1-PAK1 axis, the final common pathway of multiple immune signaling cascades that may predict patient response to anti-TNFα agents in both IBD and rheumatoid arthritis (RA) using cell-centered individual-level disruption networks, a computational method to examine high-throughput blood omics. Patients were profiled by gene expression, cytometry by time of flight (CyTOF), and Luminex before, during, and after the initiation of treatment. Using publicly available gene expression datasets from peripheral blood samples from disease-free individuals and patients with IBD in various disease states, the authors constructed a reference IBD axis using the external dataset and used a principal component analysis (PCA) plot to show the molecular transition between active disease, disease in remission, and disease-free groups on an “inflammatory axis.” The results reveal that responders progress toward the disease-free reference samples as early as week 2 after the initiation of treatment, while non-responders regress toward diseased sample data points. This novel approach allows for the contextualization of molecular changes in peripheral blood throughout treatment by positioning patients along an inflammatory axis at the individual level and using progression or regression along the axis to inform therapeutic decision-making.

To identify cellular changes following treatment in each response group, the authors have characterized immune cell composition to demonstrate variance of cell composition in responders when compared to non-responders as early as week 2, with decreased innate immune cells (such as monocytes, granulocytes, and regulatory T cells) and an increased abundance of CD4^+^ and CD8^+^ effector memory T cells and B cells. Subsequent cell-centered co-expression network and functional enrichment analyses revealed multiple transcriptional pathways related to the innate immune response to be altered, including, but not limited to, Toll-like receptors, cytoskeleton organization, FC receptor signaling, and phagocytosis. However, as the authors point out, it is crucial to emphasize that the correlation observed between gene expression levels and cell abundances does not necessarily imply that these cells are solely responsible for the observed changes in gene expression in peripheral blood. To overcome this limitation, single-cell RNA sequencing (scRNA-seq) was used to further elucidate the cellular sources of the observed changes. The use of scRNA-seq in this work to validate the gene regulatory networks highlighted by the co-expression network and function enrichment analyses further strengthens the molecular findings highlighted by the authors. It serves as proof of concept for the utility of aggregating data from multiple omic modalities to power discovery.

The methods described in this work by Gerassy-Vainberg et al. allow us to examine high-throughput molecular data at both an individual patient level and an individual cell level. Furthermore, the models they create also contextualize disease response in a cell-centered manner by examining changes in cellular composition and transcriptional regulation. Access to large, well-annotated datasets must be maintained to ensure the rigor, reproducibility, and generalizability of studies utilizing multimodal data. Additionally, it is important to highlight a large limitation of in-depth omic generation in the present: a lack of longitudinal clinical, social, environmental, and behavioral data collection regarding patient compliance, social determinants of health, and barriers to treatment response. Thus, it is important to consider that the often-clinical outcome may not lie in multiple layers of genomics, metabolomics, or proteomics but in something as simple as non-adherence. In short, as we collect data representing the depth of an individual’s biology, we must also consider the breadth of interactions between that individual and their environment.

The disruption in the RAC1-PAK1 in non-responders represents only a fraction of the findings in this manuscript—the integration of multiple molecular and computational techniques in a biologically contextualized manner represents an innovative step forward into the realm of precision medicine-based diagnostic and prognostic tools in IBD and beyond.
